# Medical Appointments

**Published:** 1847-11

**Authors:** 


*Medical Appointments.*—Prof. John P. Harrison has been transferred to the chair of Theory and Practice in the Medical College of Ohio. He is succeeded in the chair of Materia Medica by Prof. L. M. Lawson, late of Transylvania University. Dr. Jas. B. Rogers, Prof. in the Franklin Med. College, of Philadelphia, has been elected to succeed Dr. Hare in the University of Pennsylvania. The vacancy in the chair of Surgery, in the Richmond Medical College, has been filled by the appointment of Dr. Charles Bell Gibson, Prof. of Surgery in the Washington Medical College of Baltimore. Dr. Jacob Randolph has been appointed Prof. of Clinical Surgery in the Med. Department of the University of Pennsylvania.

